# Spasticity, Motor Recovery, and Neural Plasticity after Stroke

**DOI:** 10.3389/fneur.2017.00120

**Published:** 2017-04-03

**Authors:** Sheng Li

**Affiliations:** ^1^Department of Physical Medicine and Rehabilitation, University of Texas Health Science Center, Houston, TX, USA; ^2^TIRR Memorial Hermann Research Center, TIRR Memorial Hermann Hospital, Houston, TX, USA

**Keywords:** spasticity, motor recovery, stroke, neuroplasticity, rehabilitation

## Abstract

Spasticity and weakness (spastic paresis) are the primary motor impairments after stroke and impose significant challenges for treatment and patient care. Spasticity emerges and disappears in the course of complete motor recovery. Spasticity and motor recovery are both related to neural plasticity after stroke. However, the relation between the two remains poorly understood among clinicians and researchers. Recovery of strength and motor function is mainly attributed to cortical plastic reorganization in the early recovery phase, while reticulospinal (RS) hyperexcitability as a result of maladaptive plasticity, is the most plausible mechanism for poststroke spasticity. It is important to differentiate and understand that motor recovery and spasticity have different underlying mechanisms. Facilitation and modulation of neural plasticity through rehabilitative strategies, such as early interventions with repetitive goal-oriented intensive therapy, appropriate non-invasive brain stimulation, and pharmacological agents, are the keys to promote motor recovery. Individualized rehabilitation protocols could be developed to utilize or avoid the maladaptive plasticity, such as RS hyperexcitability, in the course of motor recovery. Aggressive and appropriate spasticity management with botulinum toxin therapy is an example of how to create a transient plastic state of the neuromotor system that allows motor re-learning and recovery in chronic stages.

## Introduction

According to the CDC, approximately 800,000 people have a stroke every year in the United States. The continued care of seven million stroke survivors costs the nation approximately $38.6 billion annually. Spasticity and weakness (i.e., spastic paresis) are the primary motor impairments and impose significant challenges for patient care. Weakness is the primary contributor to impairment in chronic stroke (1). Spasticity is present in about 20–40% stroke survivors (2). Spasticity not only has downstream effects on the patient’s quality of life but also lays substantial burdens on the caregivers and society (2).

Clinically, poststroke spasticity is easily recognized as a phenomenon of velocity-dependent increase in tonic stretch reflexes (“muscle tone”) with exaggerated tendon jerks, resulting from hyperexcitability of the stretch reflex (3). Though underlying mechanisms of spasticity remain poorly understood, it is well accepted that there is hyperexcitability of the stretch reflex in spasticity (4–7). Accumulated evidence from animal (8) and human studies (9–18) supports supraspinal origins of stretch reflex hyperexcitability. In particular, reticulospinal (RS) hyperexcitability resulted from loss of balanced inhibitory, and excitatory descending RS projections after stroke is the most plausible mechanism for poststroke spasticity (19). On the other hand, animal studies have strongly supported the possible role of RS pathways in motor recovery (20–36), while recent studies with stroke survivors have demonstrated that RS pathways may not always be beneficial (37, 38). The relation between spasticity and motor recovery and the role of plastic changes after stroke in this relation, particularly RS hyperexcitability, remain poorly understood among clinicians and researchers. Thus, management of spasticity and facilitation of motor recovery remain clinical challenges. This review is organized into the following sessions to understand this relation and its implication in clinical management.

- Poststroke spasticity and motor recovery are mediated by different mechanisms
- Motor recovery are mediated by cortical plastic reorganizations (spontaneous or *via* intervention)
- Reticulospinal hyperexcitability as a result of maladaptive plastic changes is the most plausible mechanism for spasticity
- Possible roles of RS hyperexcitability in motor recovery
- An example of spasticity reduction for facilitation of motor recovery

## Poststroke Spasticity and Motor Recovery are Mediated by Different Mechanisms

In the course of complete motor recovery, motor recovery follows a relatively predictable pattern regardless of stoke types (hemorrhagic or ischemic, cortical or subcortical) (39). Brunnstrom (40, 41) empirically described the stereotypical stages of motor recovery: (1) flaccidity; (2) appearance of spasticity; (3) increased spasticity with synergistic voluntary movement; (4) movement patterns out of synergy and spasticity begins to decrease; (5) more complex movements and spasticity continues to decrease; (6) spasticity disappears; and (7) full recovery of normal function with coordinated voluntary movements. Broadly speaking, there are three recovery stages: flaccid, spastic (emerging, worsening, and decreasing, stages 2–5), and recovered (voluntary control without spasticity, stages 6–7). During the course of motor recovery, stroke survivors could progress from one recovery stage to the next at variable rates, but always in an orderly fashion and without omitting any stage. However, recovery may be arrested at any one of these stages (39, 41). The classification of motor recovery stages is well accepted and used in clinical practice. The pattern of motor recovery and spasticity is confirmed in a recent longitudinal study in 2011 (42).

It is commonly observed that hyperreflexia and spasticity are gradually developed after stroke. There is no sudden change to hyperreflexia (43). The emergence of spasticity, though highly variable (44), is usually seen between 1 and 6 weeks after the initial injury (45). This implies that the development of poststroke spasticity is related to neuronal plastic changes within the central nervous system after the initial injury [see reviews (4–7, 45–47)]. Intensive therapy improves motor function, but has no effect on spasticity (48). A single dose of selective serotonin reuptake inhibitors (10 mg escitalopram) significantly increased spasticity (measured by reflex torque) without affecting muscle strength of spastic leg muscles after stroke (49). In contrast, another study (50) showed that cyproheptadine, an anti-serotonergic agent, helped reduction of muscle relaxation time possibly *via* reduction of RS excitability and spasticity reduction in the finger flexors, but without affecting muscle strength in spastic hand muscles after stroke. These findings indicate that (1) spasticity and motor recovery are mediated by different mechanisms; (2) the development of spasticity is a milestone in the course of recovery, but reflects a phenomenon of abnormal plasticity; and (3) In chronic stroke, motor recovery is arrested or plateaued. Different stages of motor recovery in chronic stroke could reflect different underlying pathophysiology in the course of motor recovery and spasticity.

## Motor Recovery are Mediated by Cortical Plastic Reorganizations (Spontaneous or *via* Intervention)

Plastic reorganization occurs immediately after stroke. Following focal damage to the motor cortex and its descending pathways, the surviving portions of the brain usually undergo substantial structural and functional reorganization that occurs in the peri-lesional areas, as well as in the ipsilesional and contralesional cortices in an animal study (51), and human neuroimaging studies (52–66). These plastic changes reflect the capability of the brain, particularly the cerebral cortex, to alter the structure and function of neurons and their networks in response to damage caused by stroke. As such, neural plasticity provides a foundation for recovery of motor function after stroke (67, 68). Motor rehabilitation relies on a combination of recovery and compensation through spontaneous recovery and motor learning during rehabilitation. True motor recovery means that undamaged brain regions generate commands to the same muscles to produce the same motor patterns, while motor compensation refers to new motor patterns (different muscles) that are controlled by alternative brain areas to accomplish the task goal (69, 70). Longitudinal studies have shown that motor recovery from hemiparesis proceeds through a series of fairly predictable stages over the first 6 months after stroke, regardless of the type of therapeutic intervention (71). During this period, there is a process of spontaneous recovery which peaks approximately in the first 4 weeks and then tapers off over 6 months. However, this does not impose physiological limits in recovery. Through novel rehabilitation protocols and mass practice, considerable motor improvement could be realized in the chronic stages (>1 year) (72). Such motor rehabilitation programs should include repetitive and task-specific practice at high intensity in a multidisciplinary environment to promote neural plasticity for motor recovery (73, 74). These motor training protocols could be realized by a number of novel neurorehabilitation methods, such as constraint-induced movement therapy (CIMT) (75, 76), robotic training (77–79), and body weight-supported treadmill training (80, 81). Accumulated evidence has supported the idea that the recovery-related cortical plastic reorganization and activation changes after the above training methods are used in chronic stroke (57, 82–85). Pharmacological agent, e.g., early prescription of fluoxetine, with physical therapy in the FLAME trial has shown to enhance motor recovery after stroke *via* modulation of spontaneous neural plasticity (86).

Both ipsilesional and contralesional motor cortices undergo plastic reorganization following a stroke, as mentioned above. Activation of bilateral sensorimotor cortices during voluntary movement of the paretic hand in stroke patients was reported (87). Activation of the contralesional hemisphere is greater in patients with poor motor function (88, 89), but decreases over time with motor recovery (57). Such changes result in abnormal interhemispheric interaction. Specifically, there is an abnormally high inhibitory drive from the contralesional hemisphere to the ipsilesional hemisphere (90). This abnormal interhemispheric inhibition correlates negatively with motor function in stroke patients. It is viewed as maladaptive plasticity (91). Based on the interhemispheric competition model, two main strategies of modulation of motor cortex excitability using non-invasive brain stimulation have been used to restore the balance of interhemispheric inhibition between lesioned and contralesional hemispheres, i.e., upregulation of excitability of the motor cortex of the lesioned hemisphere and downregulation of excitability of the motor cortex in the contralesional hemisphere (92). Restoration of interhemispheric inhibition *via* tDCS (58, 93) or rTMS (59, 94, 95) has shown to facilitate recovery of motor function in stroke patients (96).

## RS Hyperexcitability as a Result of Maladaptive Plastic Changes is the Most Plausible Mechanism for Spasticity

Spasticity is resulted from hyperexcitability of the stretch reflex, which is gradually developed after stroke (4–7). It is attributed to disinhibition of stretch reflexes as a result of altered descending inputs to spinal stretch reflex circuits after stroke (97). Disruption of descending supraspinal inputs after stroke could lead to plastic rearrangement at segmental levels (4, 5, 7, 98). In a recent animal study, Sist et al. (98) have demonstrated that there is a time-limited period of heightened poststroke structural plasticity in both brain and spinal cord after a sensorimotor stroke. The spinal plastic change correlates with the severity of cortical injury.

Excitability of the stretch reflex circuit (afferent fibers, spinal motor neurons, and efferent fibers) is predominantly regulated by excitatory and inhibitory descending signals of supraspinal origins (4, 6, 7, 99, 100). In a neurologically intact person, the descending reticulospinal tract (RST) and vestibulospinal tract (VST) provide a balanced excitatory and inhibitory descending regulation. Other descending pathways are either not related to the spinal stretch reflex (corticospinal and tectospinal) (6, 8, 100) or absent in humans (rubrospinal tract) (101). Dorsal RST descends in parallel with CST in the dorsolateral funiculus and provides a dominant inhibitory effect on the spinal stretch reflex, while medial RST and VST descend in the ventromedial cord, providing excitatory inputs. It is important to note that dorsal RST receives facilitation from the motor cortex *via* corticoreticular projections, which run in close proximity with the corticospinal tract. In stroke with cortical and internal capsular lesions, damages often happen to both CST and corticoreticular tracts due to their anatomical proximity, resulting in loss of cortical facilitatory input to the medullary inhibitory center, thus less inhibition from dorsal RST. This leaves the facilitatory medial RST and VST unopposed, since they are independent of cortical control, thus the stretch reflex hyperexcitability [see Figure 2 in Ref. (19)]. This mechanism could also explain why a stereotyped pattern of spasticity is observed regardless of affected areas (cortical or subcortical stroke).

There is experimental evidence from animal and human studies to support the important role of RST in spasticity [reviewed in Ref. (6, 8, 100)]. For example, surgical section of unilateral or bilateral VST in the anterior cord has little effect (102) or a transient effect (103) on spasticity. With more extensive cordotomies that damaged the medial RST, spasticity was drastically reduced (103). Given unilateral nature of vestibulospinal projections (104), the role of VST in spasticity was recently tested in chronic stroke (105). Vestibular-evoked myogenic potentials in the sternocleidomastoid muscle in response to high-level acoustic stimuli (130 dB) to the ears of stroke survivors were greater on the impaired side than the non-impaired side. There existed a strong positive relationship between the degree of asymmetry and the overall severity of spasticity from upper and lower limbs in spastic-paretic stroke survivors. The findings thus suggest a possible role of hyperexcitability of VST in poststroke spasticity (105). Yet, this level of acoustic stimuli is also likely to activate RS pathways *via* acoustic startle reflex (ASR) (106, 107).

Acoustic startle reflex has been used to examine RS excitability non-invasively in stroke survivors (17, 18, 108–111). In stroke survivors with cerebral infarcts normal, ASR responses could be elicited in flaccid muscles in the acute phase, although no muscle response to magnetic cortical stimulation of the primary motor cortex was elicited in these subjects (108). This suggests that the circuit of ASR remained intact in these patients. In chronic stroke, exaggerated ASR responses were observed in spastic muscles (109), indicating increased RS excitability. In a recent study (17, 18), ASR responses were examined in chronic stroke at different stages of motor recovery (flaccid, spastic, and recovered). Exaggerated ASR responses were observed only in spastic biceps muscles. Since motor recovery has been arrested in chronic stage, such findings support the important role of RS hyperexcitability in mediating poststroke spasticity. Given its role in maintaining joint position and posture against gravity (112), RS hyperexcitability and its anti-gravity effect is expected to lead to a new neuromuscular balance, reflecting a shift in reference configuration after stroke (113, 114). This new balance could be reflected by a change in the resting angle of a joint. Bhadane et al. recently found that there were strong correlations between the resting angle of the elbow joint and severity of spasticity as reflected by clinical (MAS and Tardieu R1 angle) and biomechanical (reflex torque) measurements (115). Pharmacological agents acting on serotonin, the primary neurotransmitter for RS pathways, could either increase (49) or decrease (50) spasticity. Collectively, emerging evidence supports the important role of RS hyperexcitability in poststroke spasticity.

## Possible Roles of RS Hyperexcitability in Motor Recovery

Contributions to motor recovery from ipsilesional and contralesional cortical reorganization through spontaneous recovery and facilitation and modulation of cortical plasticity are well recognized, as stated above. In contrast, RS hyperexcitability has been viewed consistently to play a major role in spasticity from both animal and human studies. The role of neural plasticity at the subcortical and bulbospinal pathways in motor recovery has been suggested from animal studies but remains controversial in human studies. In general, recovery of motor function after stroke depends on structural integrity, including both CST and RST (66, 116–118).

Findings from recent animal studies suggest the potential role of existing descending bulbospinal pathways, particularly RS projections to spinal interneurons and motoneurons (23, 26–29, 36). Riddle and Baker (29) reported that RS (descending from medial brainstem) and corticospinal pathways descended in parallel and had largely overlapping effects on spinal interneurons and motoneurons; importantly, responses from spinal motoneurons to stimulation of either pathway at supraspinal levels were of similar amplitudes during a reach and grasp task. The findings suggest the important role of RST in the distal limb muscles, in addition to its known contribution to proximal limb muscles (30). Buford and colleagues also reported significant RS contributions to motor output (35) and motor recovery (36). The rubrospinal tract descending from the lateral brainstem is almost absent in humans (101). In the context of damage to M1 and/or corticospinal pathways, strengthening the existing intact RS projections is thus plausible to compensate for the damage as demonstrated in these animal models (29, 32, 33, 35, 36).

The possible role of RS pathways in motor recovery after the corticospinal (CST) damage as result of a stroke in humans has been controversial (37, 38). Recently, Byblow and colleagues recommended that the importance of the cortico-reticulo-spinal pathway needs to be considered before using non-invasive brain stimulation to suppress contralesional motor cortex excitability because it may contribute to motor recovery, particularly in patients with severe paresis (37). However, they agreed with previous reports (58, 59, 62, 63) that suppression of contralesional cortical excitability is beneficial for those with less motor impairment. This view is further supported by findings of another recent study (38). Auditory stimulation improves motor performance of wrist extension in chronic stroke patients with spasticity and severe paresis (spastic paresis), but not in patients with more spasticity and relatively less paresis (spastic co-contraction) or with minimal paresis. The main mechanism is thought to be stimulation of RS pathway *via* auditory stimulation (38, 119, 120). Taken together, these studies in stroke survivors suggest that RS hyperexcitability and spasticity are phenomena of maladaptive changes in the course of motor recovery (19), and the role of RS hyperexcitability depends on the severity of motor impairments.

The findings (38) further suggest that RS pathway plays different roles at different stages of motor recovery, likely because of its potential role in spasticity after stroke. Individualized rehabilitation protocols utilizing RS pathways could be developed to facilitate motor recovery in some patients. In patients with severe motor impairment and spasticity, RS pathway activation *via* auditory stimulation training (38) may contribute to gross motor strength *via* synergistic activation (121), thus improving motor performance. However, such synergistic activation is not likely to improve performance of isolated wrist extension in patients with spastic co-contraction in both wrist flexors and extensors or in patients without spasticity (38). Furthermore, motor recovery after stroke follows a predictable pattern, from flaccid to spastic and to recovered stages. Auditory stimulation training *via* activation of the RS pathway (rhythmic cueing, music therapy, etc.) (38, 122–125) may be recommended for use in patients with severe motor impairment and in acute and subacute phases; as such, this intervention could potentially facilitate the progress of motor recovery after stroke, i.e., moving through the recovery stages faster in some patients.

## An Example of Spasticity Reduction for Facilitation of Motor Recovery

Spasticity is an important milestone in the course of motor recovery. It emerges and disappears as the recovery progresses. In chronic stroke when motor recovery is plateaued or arrested, e.g., spastic stages (Brunnstrom stages 2–5), spasticity usually leads to synergistic patterns of abnormal movement and impaired motor control (39, 41, 126). A stroke survivor actually flexes the fingers in an attempt of voluntary finger extension, due to abnormal co-activation of spastic finger flexors overriding weak finger extensor muscles (127). In a study examining arm pointing movements to different targets on a horizontal surface, Levin reported that stroke subjects with severe spasticity were able to plan and move the arm to all parts of available workspace, but their actual movement was deviated from smooth straight lines with increased dispersion and segmentation (128). The results demonstrate deficits in inter-joint coordination of activation of spastic muscles in spastic stroke survivors. Hemiplegic stroke survivors could accurately perceive and reproduce a force within a limb either by the spastic-paretic limb or contralateral limb (129). Force produced by one limb could not be accurately perceived by the contralateral limb in hemiplegic stroke survivors (130). Interactions between two limbs are altered (17, 18, 131). Impaired motor control in spastic stroke survivors is related to spontaneous firing of motor units and involuntary control of activation of spastic muscles (13, 14, 16), possibly caused by RS hyperexcitability (19). On the other hand, it is also important to point out that spasticity could be beneficial in the lower extremity. For example, spasticity in quadriceps may help stabilize the knee joint during the stance phase and thus help transfers.

Understanding of these two separate mechanisms underlying motor recovery and spasticity and of the role of spasticity in impaired motor control is critical for its successful management. Aggressive management of spasticity with botulinum toxin (BoNT) in carefully selected muscles can purposefully reduce involuntary activation of spastic muscles, thus to improve voluntary control of movement and motor function. BoNT blocks the release of acetylcholine presynaptically at the neuromuscular junction and transiently weakens the muscle (132). BoNT injection induces synapse plasticity of muscular afferents and generates synaptic plastic reorganization at spinal motor neurons and interneuron system and beyond. As such, the central effect of BoNT therapy converts the neuromotor system into a transient labile state (133). This allows regrowth or strengthening of appropriate synapses and suppression of inappropriate ones, i.e., neural plasticity and motor re-learning, if coupled with sustained activity-based, goal-oriented training programs (134). This is particularly important for motor recovery in chronic stroke when motor recovery is usually plateaued or arrested. For example, injection of BoNT to spastic finger flexors weakens grip strength as expected, however, the patient is able to release her grip better with decreased co-activation from finger flexors and, therefore, to engage the spastic-paretic hand more in bimanual tasks (135). Similarly, suppression of involuntary activation of periscapular muscles improves arm function and thus activities of daily living (136). This concept of “therapeutic weakness” is further supported by a recent study (137). After BoNT injection to elbow, wrist, and finger flexors, spastic hemiparetic stroke survivors are able to perform reaching (elbow and wrist extension) tasks better. The authors have attributed this functional improvement to better voluntary control of antagonists (extensors), despite of weakness of injected flexors.

## Concluding Remarks

Neural plasticity is an important process mediating substantial recovery of motor function after stroke. However, some changes may be maladaptive. The RS hyperexcitability is the most plausible mechanism for spasticity, while recovery of strength and motor function is mainly related to cortical reorganization. It is important to differentiate and understand that motor recovery and spasticity have different mechanisms. Facilitation and modulation of neural plasticity through rehabilitative strategies, such as early interventions with repetitive goal-oriented intensive therapy, appropriate non-invasive brain stimulation, and pharmacological agents are the keys to promote motor recovery after stroke. Individualized rehabilitation protocols could be developed to utilize or avoid the maladaptive plasticity, such as RS hyperexcitability in the course of motor recovery. Aggressive and appropriate spasticity management with BoNT therapy is an example of how to create a transient plastic state of the neuromotor system that allows motor re-learning and recovery in chronic stages.

## Author Contributions

The author confirms being the sole contributor of this work and approved it for publication.

## Conflict of Interest Statement

The author declares that the research was conducted in the absence of any commercial or financial relationships that could be construed as a potential conflict of interest.
